# COVID-19 associated liver injury: An updated review on the mechanisms and management of risk groups^☆^

**DOI:** 10.1016/j.livres.2023.07.001

**Published:** 2023-07-13

**Authors:** Yue Shi, Mina Wang, Liqun Wu, Xuexin Li, Zehuan Liao

**Affiliations:** a Second Clinical Medical College, Beijing University of Chinese Medicine, Beijing, China; b Graduate School, Beijing University of Chinese Medicine, Beijing, China; c Department of Acupuncture and Moxibustion, Beijing Hospital of Traditional Chinese Medicine, Capital Medical University, Beijing Key Laboratory of Acupuncture Neuromodulation, Beijing, China; d Department of Medical Biochemistry and Biophysics, Karolinska Institutet, Solna, Sweden; e School of Biological Sciences, Nanyang Technological University, Singapore, Singapore; f Department of Microbiology, Tumor and Cell Biology (MTC), Karolinska Institutet, Stockholm, Sweden

**Keywords:** Coronavirus disease 2019 (COVID-19), Liver injury, Severe acute respiratory syndrome coronavirus 2 (SARS-CoV-2), Risk groups, Treatments, Management

## Abstract

Coronavirus disease 2019 (COVID-19) has been associated with various liver injury cases worldwide. To date, the prevalence, mechanism, clinical manifestations, diagnosis, and outcomes of COVID-19-induced liver injury in various at-risk groups are not well defined. Liver injury may arise in the prevention and treatment of COVID-19 from direct causes such as viral infection and indirect causes such as systemic inflammation, hypoxic changes, and drugs that exacerbate any pre-existing liver disease. Studies have found that patients with underlying liver disease are at higher risk of COVID-19-induced liver injury. Certain condition of cardiopulmonary and metabolic diseases and vulnerable stages in lifespan may also involve in the development of COVID-19-induced liver injury. This review summarized studies of COVID-19-induced liver injury in different at-risk groups regarding their clinical characteristics, parameters, and correlations of the severity with these indicators and signs as well as potential treatment suggestions, to increase attention to physiological and pathological conditions and continue liver function monitoring as they can help in strengthening early supportive treatment and reducing the incidence of adverse outcomes.

## 1 Introduction

Since December 2019, coronavirus disease 2019 (COVID-19) caused by severe acute respiratory syndrome coronavirus 2 (SARS-CoV-2) has become a global issue that threatens public health and presents a huge challenge to health systems.^1^, ^2^, ^3^, ^4^ Various liver function abnormalities can occur in patients with COVID-19. COVID-19-induced liver injury is defined as any liver damage appearing in the infection course and treatment, regardless of whether the patient has pre-existing liver disease.^5^ Generally, 10.5%–69.0% of hospitalized patients infected by COVID-19 presented liver function examination abnormality,^1^ and in China, 24.7% of patients in the early stage of COVID-19 suffered from liver injury.^6^

Although COVID-19-associated liver injury is generally mild, transient, and self-relieved, hepatic dysfunction may lead to multisystem manifestations in the presence of acute phase reactants and coagulation factors.^5^^,^^7^ For instance, abnormal liver function was found in 29% of the deaths according to a retrospective analysis.^8^ Alternatively, liver dysfunction potentially induces continuous lung injury, which is the most prevalent death course in COVID-19.^9^ Furthermore, patients with severe COVID-19 infection were more likely to suffer from severe liver injury compared with patients with mild COVID-19.^1^^,^^10^, ^11^, ^12^ In addition, since the primary metabolic and detoxifying organ is the liver, the safety and efficacy of antiviral drugs metabolized in the liver can be even affected by a moderate loss of liver function.

In COVID-19 infection, it should be determined whether the liver injury is due to a direct effect of the virus, immune response dysregulation, drugs, pre-existing liver disease, or a complicated disease course.^13^ Thus, this review highlights recent studies that proposed groups potentially at risk of liver injury among people with pre-existing diseases or under certain physical conditions. Understanding these groups potentially at risk of liver injury can be crucial for clinical decision and outcome optimization in the management of COVID-19.

## 2 Overview of mechanisms

**Fig. 1:**
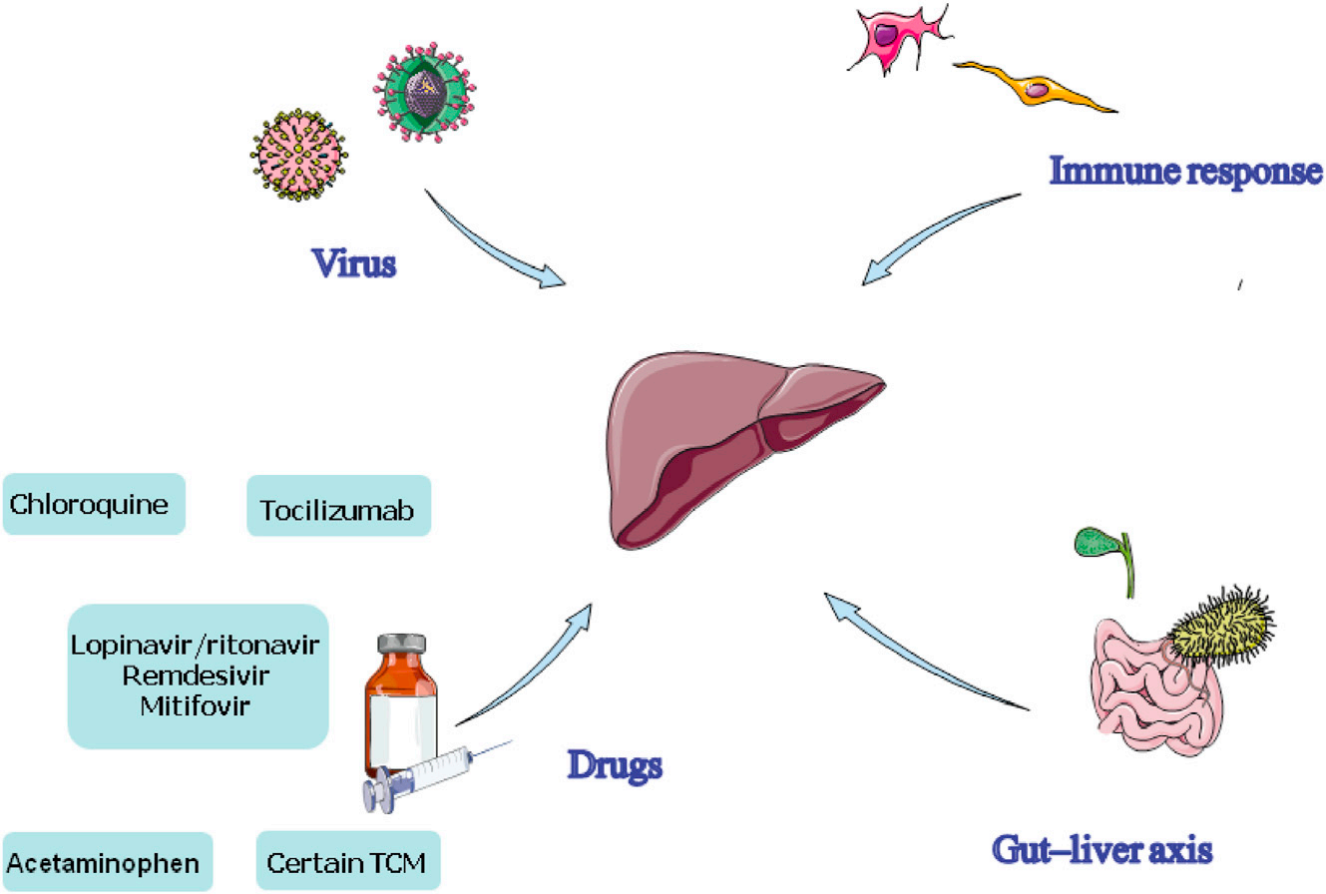
**Mechanism of COVID-19-induced liver injury.** This figure demonstrates several mechanisms of liver injury related to COVID-19, including direct injury caused by the virus, activated immune response, suspicious drug usage (anti-virus drugs, tocilizumab, chloroquine, certain TCM, and acetaminophen), and infection in the gut–liver axis. Abbreviations: COVID-19, coronavirus disease 2019; TCM, traditional Chinese medicine.

As shown in Fig. 1, several mechanisms have been proposed to explain how liver injury occurs during COVID-19. Cross-group mechanisms are discussed in this section, whereas specific mechanisms will be presented in different risk groups.

### 2.1 Direct injury caused by the virus

The liver plays an essential role in defending microbes and is involved in systemic infections for the reception of the portal and systemic circulation.^13^ Dong *et al*.^14^ suggested that viral RNA could be detected in the liver tissue, which contributes to the hypothesis of virus-mediated liver injury. Furthermore, Schattenberg *et al*.^15^ stated that virus-mediated injury to cholangiocytes, which are crucial for hepatocyte homeostasis, may also cause liver cell injury.

Establishment of SARS-CoV-2 infection in humans occurs with the interaction of angiotensin-converting enzyme 2 (ACE2) receptor,^16^ which is minimally expressed on hepatocyte membranes in normal controls, and could be observed in most liver injury cases. Previous studies have also found that strains with greater infectivity (B.1.1.7, B.1.351, P.1, and B.1.617.2) may have a high affinity for ACE2 and express multiple mutations of the S protein,^17^, ^18^, ^19^, ^20^ a structural protein of SARS-CoV-2 for its entry into the host cells. Receptor binding is performed by the S1 subunit,^21^ and the receptor-binding domain (RBD) binds to ACE2,^22^ whereas membrane fusion is accomplished by the S2 subunit.^21^ The viral RNA enters the hepatocytes expressing low levels of ACE2 and TMPRSS2 through membrane fusion.^23^ First, ACE2 combines with RBD to change the conformation of the S protein. Meanwhile, furin protease recognizes and cleaves the polybase insertion site in S1/S2 to induce S1/S2 subunit dissociation,^24^ which changes the conformation of the S2 subunit to expose the S2′ cleavage site cleaved by TMPRSS2. Subsequently, the exposed fusion peptide is inserted into the target cell membrane,^25^ binding the viral and cellular membranes together to form a fusion pore for RNA release.^26^ At the metabolic level, SARS-CoV-2-infected hepatocytes have impaired glucose and glutamine metabolism, which is mediated in part by inhibiting the renin–angiotensin system (RAS) through the interaction between the S protein and ACE2. The enhancement of glutaminolysis metabolism with the upregulation of glutamate levels triggers the replication of SARS-CoV-2.^27^

Generally, since ACE2 regulates energy metabolism, protecting against oxidative stress and inflammation, the hypothesis that ACE2 depletion resulting from SARS-CoV-2 infection contributes to metabolic dysfunction and inflammation may be reasonable.^28^ Particularly, SARS-CoV-2 compromises mitochondrial activity and impairs energy metabolism. The depletion of the angiotensin-receptor Mas in hepatocytes aggravated mitochondrial dysfunction, increased mitochondrial reactive oxygen species (ROS), induced fatty acid synthesis, and impaired cholesterol synthesis/efflux.^29^ Furthermore, increased iron uptake in the presence of a viral infection triggers mitochondrial ROS.^30^ This imbalance in iron metabolism and mitochondrial ROS production may result from the alteration of cellular oxidative homeostasis promoted by an exacerbated mitochondrial activity in infected hepatocytes, leading to an apoptotic response and liver injury.^31^ Alternately, the infected hepatocytes present a glycolytic phenotype characterized by increased production of lactic acid and a more active tricarboxylic acid cycle. This altered glycolytic metabolism supports viral replication in the liver and contributes to evading cytotoxic immune responses by acidifying the extracellular compartment.^31^ These findings confirm that SARS-CoV-2 enters into the hepatocytes by binding to ACE2, which is responsible for subsequent histological injury.

### 2.2 Activated immune response

The dysregulation of the innate immune response, which exists in hepatic inflammation, may lead to liver injury in COVID-19.^13^ COVID-19 may induce a severe inflammatory response that is characterized by a significant increase in the levels of cytokines and chemokines released by the activated immune cells, which is known as a cytokine storm or cytokine release syndrome.^32^ These cytokines induce original hepatic stellate cells to transdifferentiate into collagen-producing myofibroblasts, deposit extracellular matrix, and secrete pro-inflammatory cytokines, which further contribute to the cytokine storm.^33^^,^^34^ This immune activation reflects liver inflammation, and the released cytokines disrupt the microenvironment in the liver such as increased triglyceride accumulation, contributing to progressive liver injury.^15^^,^^35^ Particularly, interleukin (IL)-6 in cytokine storm plays a key role in liver injury, which stimulates hepatocytes during the initial phase of inflammation to upregulate C-reactive protein (CRP), fibrinogen, haptoglobin, alpha-antitrypsin, and serum amyloid-A, which induce an acute inflammatory phase.^36^ Alternately, prolonged inflammation stimulates IL-6, targeting monocyte chemotaxis toward tissue-destructive injury.^37^ Moreover, viral-specific CD8^+^ T cells generated in response to SARS-CoV-2 infection can trigger T cell-mediated hepatitis via the activation of Kupffer cells.^38^ Therefore, the uncontrolled pro-inflammatory cytokine release and immune cell recruitment represent unfavorable clinicopathological conditions, leading to progressive liver damage and failure.

### 2.3 Drug-induced liver injury (DILI)

The clinical benefits of anti-SARS-CoV-2 drugs are currently uncertain or controversial,^39^ in which lopinavir/ritonavir, tocilizumab, chloroquine, remdesivir, mitifovir, and certain traditional Chinese medicine may have a toxic influence on the liver in some patients.^1^^,^^40^ Zha *et al*.^41^ reported that lopinavir/ritonavir induces liver injury by aggravating liver endoplasmic reticulum stress and hepatocyte apoptosis. Cao *et al*.^42^ found that ritonavir causes liver injury because it occupies the cytochrome P450 system, which metabolizes varying toxic intermediates of different drugs. Ferrara *et al*.^43^ stated that tocilizumab inhibits the IL-6 pathway (indispensable for liver regeneration), leading to liver injury, liver failure, and liver transplant (LT) requirements. Moreover, apart from anti-SARS-CoV-2 drugs, symptom-relieving drugs may also cause liver injury. Acetaminophen is used to treat mild-to-moderate pain and fever in some patients with COVID-19, and its overdose stimulates reactive metabolite formation, which subsequently depletes glutathione and triggers mitochondrial dysfunction, leading to hepatocellular necrosis and even acute liver failure.^44^ Alternately, acetaminophen is revealed to exert a dose-dependent reduction in the expression level of ACE2 in primary hepatocytes.^45^ Since decreased ACE2 may contribute to hepatocytic pathology and acetaminophen induces a dose-dependent change in the expression of entry genes, chronic users of acetaminophen for certain diseases may be exposed to a greater risk of liver injury because of a more pronounced change.

### 2.4 Infection in the gut–liver axis

COVID-19 may also affect the gastrointestinal tract and induces liver infection by translocating hepatocytes via the portal vein.^46^ Subsequently, through transcytotic vesicular pathways, SARS-CoV-2 that existed in the hepatocytes reaches the bile. Then, the second wave of infection may occur in the biliary tract, which links the liver and the gut directly.^5^ Thus, the vicious circle provides a preferable environment for the existence of the virus, which may worsen the overall outcomes in patients with COVID-19 and hepatic and intestinal symptoms.

## 3 General inspection

Laboratory inspection, imaging examinations, and other signs may indicate the existence of liver injury. Although it appears similar in different risk groups, it may assist in the early diagnosis of pre-existing liver injury in these vulnerable groups.

Hypoalbuminemia was the most notable abnormality, which may be combined with high levels of gamma-glutamyl transferase (GGT), aminotransferases, bilirubin (BIL), and alkaline phosphatase (ALP). Moreover, Kumar-M *et al*.^47^ observed liver function abnormalities such as hypoalbuminemia and high levels of GGT, aminotransferase, and BIL in severe cases. Yu *et al*.^1^ suggested that COVID-19-associated liver injury should be identified when alanine aminotransferase (ALT) or aspartate aminotransferase (AST) elevates three times, and ALP, GGT, or BIL elevates two times over the upper limit of their normal values. Although the elevation of ALT and AST levels similarly occurred in all patients with COVID-19, the prevalence of elevated AST is often higher than ALT in severe COVID-19,^47^ which was different from the findings of Marjot *et al*.,^48^ reporting a classic hepatocellular pattern of liver injury. Moreover, Schattenberg *et al*.^15^ recognized that most patients exhibited intracellular markers of cytolysis such as lactate dehydrogenase and creatine kinase, which may indicate the extent of injury in cells. In addition, Lei *et al*.^49^ observed that some patients with COVID-19 had abnormal prothrombin time and hypoalbuminemia, which may be due to liver injury or coagulopathy. Notably, levels of cytokines such as tumor necrosis factor-alpha (TNF-α), IL-1, and IL-6 may also increase because of cytokine storm.^5^

Furthermore, radiological changes occur in the liver of patients with COVID-19-induced liver injury. According to Lei *et al*.,^49^ upper abdominal computed tomography (CT) demonstrated liver hypodensity and pericholecystic fat stranding, and the liver-to-spleen attenuation ratio and pulmonary lesions positively correlated with disease severity. According to logistic regression analysis, severe lung lesions are closely linked to liver dysfunction, indicating that the degree of lung lesions on CT may predict liver function abnormality.^1^

In addition, a study found an association between liver injury and body temperature, i.e., the peak serum levels of liver enzymes were notably related to the highest body temperature.^50^

## 4 Risk groups

**Fig. 2:**
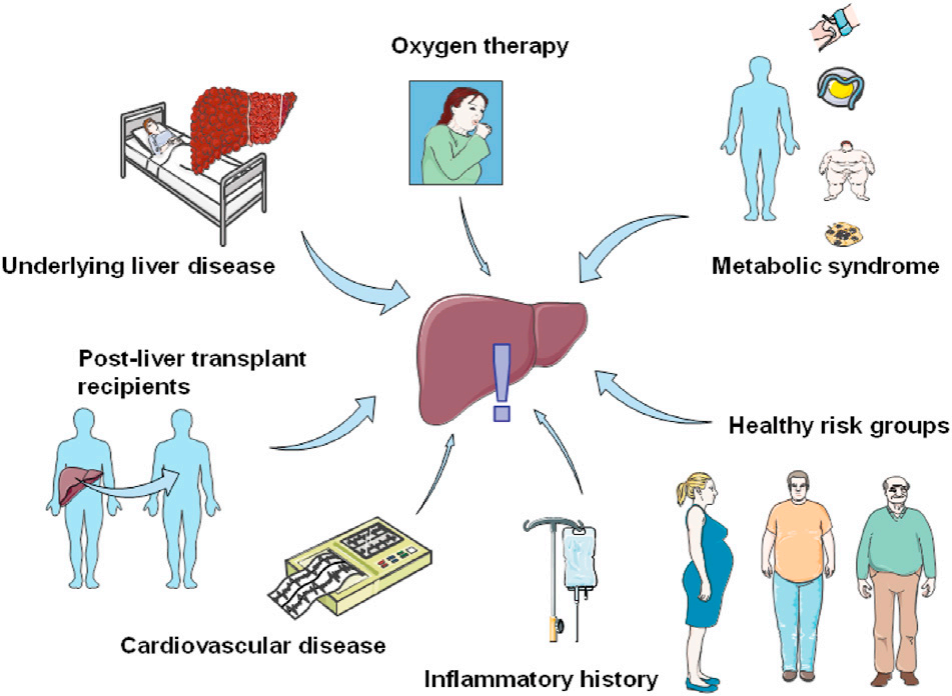
**Risk groups of coronavirus disease 2019 (COVID-19)-induced liver injury.** This figure demonstrates groups that are at risk of COVID-19-induced liver injury, including patients with pre-existing disease (chronic viral hepatitis, cirrhosis, hepatocellular carcinoma, and alcohol-associated liver disease), post-liver transplant recipients, patients with metabolic syndrome (hypertension, dyslipidemia, diabetes, and obesity), and patients with cardiovascular diseases. Patients with certain history may also be at risk, including those who are receiving oxygen therapy or have an inflammatory history. In a healthy population, older people, men, and probably pregnant women are also vulnerable groups.

Fig. 2 presents the groups that are at risk of COVID-19-induced liver injury, as summarized from the results of previous studies.

### 4.1 Risk groups in patients with pre-existing diseases

Certain groups of patients are more vulnerable to COVID-19-induced liver injury. Among all groups, the prevalence of acute liver injury was reported to be 23.70% and 31.66% in two studies, whereas it was 44.63% in patients with severe and 20.02% in those with non-severe diseases.^47^

#### 4.1.1 Pre-existing liver disease

Pre-existing liver diseases were observed in a considerable number of patients with COVID-19,^47^ and in those patients, the overall prevalence of chronic liver disease (CLD) was 2%–11%.^1^ In detail, the prevalence values of pre-existing liver disease were approximately 11% in Zhejiang and 9% in Wuhan.^13^ Among studies reporting severity, the pooled prevalence of CLD was 2.64%, with 3.03% in patients with severe and 2.20% in those with non-severe diseases.^47^ However, the role of pre-existing end-stage liver disease is still under exploration.

Patients with pre-existing liver diseases are apt to have abnormal liver function tests (LFTs). According to a multicenter study in the USA by Kumar-M *et al*.,^47^ a three-time higher proportion of AST or increased GGT levels can be observed in these patients. Particularly, Di Sessa *et al*.^51^ revealed that the overall prevalence of mild increases in liver enzymes was approximately 25% in children with COVID-19, indicating that liver abnormalities may manifest as hepatic steatosis and/or elevated liver enzymes in pediatric patients.

Therefore, in patients with pre-existing liver diseases, the pre-existing disease must be treated, hepatocellular carcinoma (HCC) and its types should be detected, and complications must be promptly managed. However, the pandemic may influence these strategies. For example, the initiation of antiviral treatment in patients with chronic viral hepatitis may be delayed, and problematic drinking may relapse, leading to the progression of pre-existing diseases and decompensating events.^48^

##### 4.1.1.1 Cirrhosis

Patients with liver cirrhosis may be more vulnerable to COVID-19, as Marjot *et al*.^52^ found high morbidity and mortality with increased severity of cirrhosis, which contributed to more prevalent invasive ventilation, renal replacement therapy, intensive care unit (ICU) admission, and death in patients with COVID-19.

In the spectrum of mechanisms, a systemic immunodeficiency status plays a crucial role by increasing the risk of infection and developing an aberrant inflammatory response, resulting in higher rates of infection and death.^53^^,^^54^ In addition, cirrhosis-related increased baseline endotoxemia and cytokine production may exaggerate inflammatory response during an infection.^48^

In the spectrum of inspection, acute hepatic decompensation was common in patients with both COVID-19 and cirrhosis, which may be the primary and only indicator of this viral infection. Similarly, acute hepatic decompensation occurs in 47% of patients with both COVID-19 and cirrhosis, of which 24% appeared without any pulmonary symptoms. Since cirrhosis grade is a mortality predictor in patients with COVID-19,^48^ typical manifestations should be identified and managed if ascites and encephalopathy aggravation are present.

In addition, impaired responses to existing approved vaccinations are common in patients with cirrhosis; however, thus far, data on the efficacy of COVID-19 vaccination in this group are lacking.^55^ Therefore, evaluating the most suitable vaccine type, dosing, or timing for patients with cirrhosis is difficult.

##### 4.1.1.2 Chronic viral hepatitis

Viral hepatitis increased the risk of patients with COVID-19 to liver injury.^9^ In the spectrum of mechanisms, viral reactivation and DILI should be emphasized. On the contrary, high-dose hormone therapy during COVID-19 may reactivate and replicate the hepatitis B virus (HBV) in patients with hepatitis B who failed in continuing anti-HBV therapy.^1^ Moreover, certain biological drugs (tocilizumab and baricitinib) may also cause HBV activation and subsequent liver function deterioration. Furthermore, Tian *et al*.^8^ suggested that combined ritonavir and lopinavir therapy may accelerate liver damage in patients with HBV or hepatitis C virus (HCV) infection. Particularly, Boeckmans *et al*.^56^ found that patients with HCV infection were at higher risk of developing a drug-induced hepatic injury when they received highly active anti-retroviral therapy.

Patients with SARS-CoV-2 and HBV co-infection tend to show a worse prognosis of liver injury.^9^ Therefore, etiologies such as hepatitis A virus, HBV, and HCV should be considered in patients with COVID-19 and enhanced liver biochemical reactions, as recommended by the American Association for the Study of Liver Diseases (AASLD).^1^

In the management of viral hepatitis during the pandemic, Yang *et al*.^57^ suggested continuous antiviral medicine therapy to prevent HBV reactivation, particularly when combined with glucocorticoids,^57^ whereas in patients with HCV, the initiation of antiviral therapy should be delayed.^1^ In addition, Meng *et al*.^58^ warned about the potentially detrimental effects of corticosteroid therapy on patients with severe COVID-19 and chronic HBV co-infection, including delayed SARS-CoV-2 clearance, increased risk of coagulopathy, and acute liver injury. Therefore, corticosteroids may be avoided in patients with COVID-19 and HBV co-infection.

##### 4.1.1.3 HCC

Patients with HCC might be more susceptible to COVID-19 because of their systemic immunocompromised status.^7^ However, Amaddeo *et al*.^59^ reported that the number of HCC diagnoses significantly reduced and the rate of HCC treatment delay doubled during the pandemic period compared with that during the prepandemic period. Since maintenance of multidisciplinary care is necessary for appropriate HCC management, telemedicine may be an option.^48^ Furthermore, large-cohort clinical studies should be conducted in patients with both HCC and COVID-19 to analyze the severity, mortality, and incidence of complications including hepatic encephalopathy, upper gastrointestinal bleeding, secondary infection, and liver failure.

##### 4.1.1.4 Alcohol-associated liver disease

Evidence on alcoholic liver disease or alcoholic hepatitis in COVID-19 is limited. However, increased mortality was reported in patients with alcohol-related cirrhosis.^9^ Notably, acetaminophen, which is prevalent in controlling high body temperatures caused by COVID-19, can also induce liver injury by activating hepatic cytochrome P450 2E1 (CYP2E1), which can occur in individuals with severe alcohol dependence.^39^

#### 4.1.2 Post-LT recipients

During the COVID-19 pandemic, LT programs have been influenced by deceased donors, risk of virus exposure and transmission, and restrained hospital resources.^60^ LT recipients are predisposed to ICU admission; although enhanced social distancing may be a protective factor, mortality rates were similar between patients with COVID-19 and healthy controls.^48^ Notably, Phipps *et al*.^61^ observed a lower risk of COVID-19-induced liver injury in non-Hispanic white LT recipients, whereas older age, Hispanic ethnicity, receipt of vasopressors, and antibiotic use increased the risk of liver injury, indicating ethnic differences in the development of liver injury. This may deserve further studies on genetic differences and public health policy to identify LT recipients with even higher risk.

Among mechanisms proposed, immunosuppression placed LT recipients at risk because of inadequate immune response against the virus and remodified immune system, which may cause acute rejection in the presence of COVID-19.^61^ In addition, hepatotoxicity should be monitored because drug–drug interactions are more likely to occur in these patients,^61^ particularly in those on taking ritonavir-boosted antiviral and immunosuppressive drugs that exhibit relevant interactions through CYP34A and result in increased calcineurin and mTOR inhibitors.^13^ Moreover, the treatment of post-LT recipients is challenging because of limited information and the vital requirement of continuous immunosuppressive therapy during the pandemic, which increases the risk of these patients to more severe COVID-19.^9^ However, some current studies have revealed that LT and associated immunosuppression may not increase the risk of undesired outcomes during the pandemic.^48^ On the contrary, the innate response may dominate the COVID-19 outcomes, which is less limited by typical immunosuppression in LT recipients.^62^ Mortality in severe COVID-19 can be reduced through active corticosteroid immunosuppression with large doses of dexamethasone.

Not all LT recipients with COVID-19 progressed into severe cases. For instance, 6% were asymptomatic in the Spanish series as reported by Colmenero *et al*.,^63^ whereas 14% were not accompanied by respiratory or gastrointestinal symptoms in a multinational series by Webb *et al*.^64^ In symptomatic cases, LT was likely related to gastrointestinal symptoms rather than respiratory features.^48^ Nevertheless, liver injury was found in 34.6% of LT recipients during the pandemic, which appeared to be a predominantly hepatocellular pattern with liver enzyme elevations.^61^ Therefore, to identify at-risk LT recipients with adverse COVID-19 outcomes early, intensive monitoring of liver enzymes may be a practical method.

Regarding prevention and treatment, the management of post-LT recipients during the pandemic is challenging because of limited data and frequent requirements for visits to healthcare facilities and continuous immunosuppressive treatment, and these patients have absolute chronic immunosuppression and frequent concurrent comorbidities, which made them more susceptible to more severe infection and potentially prolonged viral shedding.^13^ Among LT patients, the immunocompromised status must be intensively monitored to reduce complications and secondary infections.^57^ Some studies have suggested the continuation of immunosuppressant drug therapy and administration of short-term steroid therapy to reduce the severity of pneumonia.^1^ However, the position statement in the European Academy of Hepatology-European Society for Clinical Microbiology and Infectious Diseases suggested appropriate adjustment of the dose of immunosuppressants according to antiviral treatment regimens because of drug interaction in both regimens.^1^ Therefore, remdesivir and chloroquine-based regimens appear to have better safety, whereas boosted protease inhibitors are not recommended.^13^

Comorbidity plays a vital role in determining the outcomes and when comparing with advanced CLD, whereas LT was less related to undesired SARS-CoV-2 outcomes,^48^ which is supportive evidence for continuing LT programs. However, a study suggested delaying LT, except for critically ill cases.^65^ Moreover, whether bridging therapy for certain etiologies is preferred over transplantation in long-term retransplantation should be determined because of organ scarcity during the pandemic.^66^

Therefore, vaccination must be provided preferentially to this vulnerable group,^48^ which is consistent with the AASLD consensus on COVID-19 vaccination in patients with liver disease. However, the immunogenicity of COVID-19 vaccines in LT recipients and patients with autoimmune liver disease and immunosuppression should be further evaluated because the full immunization regimen of mRNA vaccine-induced humoral response appeared adequate in solid-organ transplant recipients, whereas antimetabolite immunosuppression was associated with a poor response.^67^

#### 4.1.3 Metabolic syndrome

Metabolic syndrome is a combination of multiple diseases, including hypertension, dyslipidemia, diabetes, and obesity.^68^, ^69^, ^70^, ^71^, ^72^, ^73^, ^74^ These metabolic comorbidities are closely related to non-alcoholic steatohepatitis/fatty liver and fatty liver associated with metabolic dysfunction.^9^ Patients with both metabolic syndrome and COVID-19 are more likely to experience various degrees of (even acute) liver injury than patients with COVID-19 without metabolic syndrome.^75^

Meanwhile, the pandemic has also propagated unhealthy lifestyles in this group;^76^ i.e., according to Pellegrini *et al*.,^77^ less exercise, excess calorie intake, and increased mental problems may result in weight gain of 1.5 kg on average in patients with obesity. In addition, increased alcohol consumption, particularly in periods of social isolation, is now well-recognized, which exposes patients with metabolic syndrome to a higher risk.

##### 4.1.3.1 Hypertension

Hypertension is characterized by abnormal activation of the RAS and a relatively low level of ACE2, whose disruption may aggravate COVID-19.^78^ Imoto *et al*.,^50^ recognized that SARS-CoV-2 directly binds to the ACE2 receptor, which mediates SARS-CoV-2 cellular entry,^15^ resulting in virus replication and subsequently infection of other cells.^50^ However, Li *et al*.^75^ reported contradictory observations suggesting that patients with hypertension are vulnerable to SARS-CoV-2 because the virus invades through ACE2 receptors. Moreover, as stated by Yang *et al*.,^78^ higher IL-6, CRP, and procalcitonin levels were observed in patients with both COVID-19 and hypertension, indicating a stronger systemic inflammatory response, which may affect liver metabolism, eventually leading to secondary liver injury.

##### 4.1.3.2 Dyslipidemia

Dyslipidemia showed significant correlation with COVID-19. Lu *et al*.^79^ suggested that high levels of total cholesterol may be associated with virus replication, whereas decreased levels of blood lipid may provide favorable conditions for virus particle synthesis and packaging during the viral invasion. Alternatively, Li *et al*.^75^ reported that liver lipid metabolic overload in patients with dyslipidemia and COVID-19 may increase the risk of liver injury through a synergistic effect. Moreover, the virus may directly lead to liver damage and aggravate lipid metabolic disorders because the virus replicates in noticeable quantities in the liver, which is the main site of lipid metabolism.

##### 4.1.3.3 Diabetes

Diabetes increases the risk of COVID-19. Hyperglycemia aggravates virus replication, subsequently expanding viral invasion and triggering a systemic inflammatory response, resulting in multisystemic damage or failure.^75^ As a disease related to autoimmune and chronic inflammation, diabetes is considered to cause autoimmune system dysfunction and immune cell imbalance, which may be further aggravated by COVID-19, leading to abnormal inflammatory reactions as reported by Guzmán-Flores *et al*.^80^ Accordingly, since the liver is a vital immune organ, diabetes combined with COVID-19 may aggravate the liver immunomodulatory load. Moreover, hypoglycemic drugs may cause abnormal increases in transaminase levels, such as acarbose, and may enhance immune-mediated liver injury such as metformin.

##### 4.1.3.4 Obesity

Obesity is an independent risk factor of COVID-19.^75^ Obesity is related to non-alcoholic fatty liver disease (NAFLD), which may cause chronic liver injury through an inflammatory response.^81^ Accordingly, obesity can affect the immune system, causing imbalanced regulation of the immune system that can increase the risk of an inflammatory storm in COVID-19, which may lead to multisystemic injury.^82^ Moreover, more severe (and/or more frequent) DILI and aggravated obesity-related pre-existing liver diseases can be seen in patients with COVID-19 and obesity due to the administration of certain drugs, which can be more hepatotoxic through drug–drug interactions, leading to altered lipid homeostasis, mitochondrial dysfunction, impaired activity of xenobiotic-metabolizing enzymes, and oxidative stress. Furthermore, Omarjee *et al*.^83^ proposed that DILI may promote the risk of cytokine storm in patients with severe COVID-19, and pro-inflammatory cytokines can notably aggravate hepatotoxicity possibility of numerous drugs such as antibiotics. In addition, Li *et al*.^75^ stated that impaired ventilation in obesity may cause hypoxemia, exposing liver metabolism to an anoxic environment, which can lead to liver injury. Interestingly, pediatric patients with COVID-19 tended to have a milder course and a better prognosis, probably because of a special immune response system.^51^

#### 4.1.4 Others

As regards other clinical features, Mishra *et al*.^84^ showed that the presence of cardiovascular disease independently increases the risk of liver injury, probably because of right-sided heart failure and subsequently high central venous pressure.

### 4.2 Risk groups in patients with a certain history

#### 4.2.1 Oxygen therapy

Jiang *et al*.^85^ reported that because of varying degrees of hypoxemia, more than 40% of patients with COVID-19 required oxygen therapy. Phipps *et al*.^61^ also revealed that patients who were admitted to the ICU and taking vasopressors tended to suffer from COVID-19-induced liver injury. Hypoxia and ischemia may play predominant roles in the development of liver injury in severe and critical COVID-19. Under hypoxia and ischemia, cell survival signal transduction can be inhibited by lipid and glycogen dysregulation, leading to rapid hepatocyte death. Moreover, hypoxia-induced oxidative stress response increases peroxidation products and ROS levels in respiratory distress syndrome, further initiating the release of various pro-inflammatory factors and subsequently inducing liver damage. Subsequently, the above changes may trigger hepatocyte hypoxia and ischemia, further aggravating liver injury.

#### 4.2.2 Inflammatory history

As a complication of COVID-19, Cichoz-Lach *et al*.^86^ reported that an inflammatory history may increase the incidence of liver function impairment. In the spectrum of mechanisms, inflammatory pathways that pre-exist in systematic inflammation can be overactivated in patients with COVID-19, which might contribute to extrapulmonary injuries.^13^ In addition, secondary liver injury can be induced by strong immune activation, called cytokine storm, which often leads to a sudden state of multiple organ failure.^15^ Moreover, drugs such as antibiotics and steroids may be routinely applied to treat inflammation in patients with COVID-19, which was suspected to contribute to the occurrence of liver dysfunction after hospitalization.^87^

### 4.3 Risk groups in healthy people

#### 4.3.1 Older population

Older people are apt to develop a more severe COVID-19 and worse liver function. Li *et al*.^88^ found that the median age was significantly higher among patients with COVID-19 with abnormal LFTs. Schattenberg *et al*.^15^ analyzed patients aged 70 years (mean age) and reported that 86% had comorbidities and 38% had abnormal LFTs. Abnormal LFT values support that the older age is predisposed to more severe liver injury in the presence of COVID-19,^13^ when accompanied by various poor prognostic factors of liver injury and a burden of pre-existing liver disease;^57^ thus, these patients must be monitored and should receive individualized treatment for potential liver injury.

#### 4.3.2 Male

Abnormal liver function was more common in men with COVID-19,^89^ indicating that men are more prone to liver injury than women. Furthermore, Chen *et al*.^90^ observed that older men tended to have higher rates of severe COVID-19 than younger women. And Cichoz-Lach *et al*.^86^ reported that acute liver injury was more likely to develop in younger men (<65 years old). As for the mechanism, hormonal imbalance with low testosterone concentration was noted in 66.7% of male patients, which was proposed as a reason for liver injury in COVID-19.^86^

#### 4.3.3 Pregnant women

Whether pregnancy is a risk factor for liver injury during the pandemic remains controversial. A study reported that liver injury occurs in 29.7% of pregnant patients with COVID-19,^91^ and another study reported 3.8%–11.8%,^92^ which is relatively lower than the rate of 21.5%–45.7% in the general population.^93^

As mentioned above, hyperinflammation is involved in COVID-19-related death. Therefore, the anti-inflammatory phase in the second trimester of pregnancy may be protective against inflammation in severe COVID-19. Alternatively, because of the low-grade inflammation and non-severe pneumonia, pregnant patients may receive fewer liver injury-associated drugs such as lopinavir/ritonavir.^89^

Although current evidence indicated no worsened outcomes in neonates, higher levels of inflammatory markers including polymerase chain reaction and IL-6 were observed in pregnant patients with COVID-19, which positively correlate with severe COVID-19, indicating the need for liver function monitoring in pregnant patients with COVID-19.

## 5 Suggestions

Regarding general diagnosis, liver biochemical indicators should be regularly monitored in all patients with COVID-19. Furthermore, due to the higher risk of liver injury development, patients with pre-existing liver diseases and post-LT patients should preferentially receive the test.

For general management, in COVID-19 cases presenting with mild liver biochemical abnormalities, the primary disease should be actively treated when supportive and antiviral therapies are given to reduce inflammation and viral replication and improve the immune system.^8^ In acute liver injury cases, causes should be analyzed to determine suitable measures, and markers of liver function and prothrombin activity should be closely monitored. Moreover, the use of enzyme-lowering drugs and liver-protection drugs and intensive monitoring should be employed once acute liver failure is identified.^8^ In addition, to balance intestinal microecology and prevent bacterial infections, prebiotics and probiotics should be administered.^57^ In cases of severe COVID-19 with liver injury caused by microcirculation hypoxia, ischemia, and cytokine storm, strengthened respiratory and circulatory support is necessary.^8^ In DILI, apart from conventional anti-inflammatory/hepatoprotective drugs, discontinuation or tapering of the dose of the suspected drugs was proposed, followed by the assessment of the degree of liver damage and subsequent adjustment of the treatment plan. Notably, up to now, the safety of drugs to treat COVID-19 is still unknown in patients with liver dysfunction.^94^ Therefore, drugs that can both protect the liver and treat systemic inflammation are recommended for patients with COVID-19-induced liver injury.^94^ In addition, adverse events of potential drugs for the treatment of COVID-19 should be frequently followed and assessed. Moreover, streamlined treatment and redundant types, doses, and durations of drugs are recommended to reduce the incidence of DILI.^57^

Accordingly, Table 1 summarizes the management of different conditions and benefits from the treatment in risk groups with COVID-19 in this review.

**Table 1:** Management of different conditions and benefits from the treatment in risk groups with COVID-19.

Conditions	Management	Benefits
HBV infection	• Continuous antiviral medicine	• Prevent reactivation of HBV, particularly in glucocorticoid treatment
HCV infection	• Delay the initiation of antiviral therapy	
Liver transplantation	• Monitor the immunocompromised status • Monitor liver biochemical indicators • Adjust/continue immunosuppressant drugs • Apply short-term steroid therapy • Avoid boosted protease inhibitors • Delay less emergent LT operation • Vaccination	• Reduce complications • Reduce secondary infections
Liver biochemical abnormalities	• Treat primary disease • Provide antiviral and supportive treatment	• Inhibit viral replication • Reduce inflammation • Improve immunity
Liver dysfunction	• Apply drugs with dual function • Monitor and assess adverse events of potential drugs	• Inhibit systemic inflammation • Protect liver functions
Acute liver injury	• Analyze and judge the causes • Monitor markers of liver function and PTA • Apply drugs with dual function	• Protect the liver • Lower enzyme
Acute liver failure	• Intensive monitoring • Apply prebiotics and probiotics	• Maintain the balance of intestinal microecology • Prevent bacterial infections
Cytokine storms Microcirculation ischemia Hypoxia	• Strengthen respiratory • Circulatory support	• Prevent life-threatening complications and worsening of comorbidities
Drug-induced liver injury	• Anti-inflammatory/hepatoprotective treatment • Stop/reduce suspected drugs • Assess the degree of liver damage • Adjust the treatment plan	• Alleviate liver injury

Abbreviations: COVID-19, coronavirus disease 2019; HBV, hepatitis B virus; HCV, hepatitis C virus; LT, liver transplant; PTA, prothrombin activity.

## 6 Conclusions and research perspectives

Liver injury is a notable complication of COVID-19, and this review aims to clarify the clinical features of patients with COVID-19-induced liver injury. We summarize current evidence related to groups at risk of hepatobiliary complications in the presence of COVID-19 and provide a summary of the mechanism, detecting methods, and recommendations for clinicians. Direct viral hepatotoxicity, hyperinflammation, DILI, and aggravation of pre-existing liver disease were discussed. Abnormalities of liver function, inflammation, and intracellular markers in COVID-19 appear to be prevalent but not severe generally. Risk groups of developing COVID-19-induced liver injury include patients with pre-existing liver disease, patients with metabolic diseases, and post-LT recipients, whereas older individuals, men, and pregnant women deserve special attention. Regarding treatment, clinical evaluation, continuous surveillance, and specific therapy should be considered for the potential risk groups of COVID-19-induced liver injury to achieve better outcomes of COVID-19 and pre-existing diseases.

Collectively, current evidence shows divergent at-risk groups of COVID-19-associated liver injury. However, the specific pathological mechanism of COVID-19-induced liver injury in different vulnerable groups still requires further research, and the evidence of COVID-19-induced liver injury patients with other suspicious past history is lacking. Moreover, due to the indefinite effectiveness and the potential side effects, the therapy to treat or prevent COVID-19-induced liver injury is warranted to be further verified.

## Authors’ contributions

Yue Shi and Zehuan Liao conceived the idea. Yue Shi wrote the manuscript and drew figures. Mina Wang, Liqun Wu, Xuexin Li, and Zehuan Liao reviewed and edited the manuscript critially. Liqun Wu and Zehuan Liao provided direct supervision. All authors read and approved the final manuscript.

## Declaration of competing interest

The authors declare that they have no conflicts of interest.

## References

[1] Yu D., Du Q., Yan S. (2021). Liver injury in COVID-19: clinical features and treatment management. Virol J.

[2] Liao Z., Menon D., Zhang L. (2022). Management of the COVID-19 pandemic in Singapore from 2020 to 2021: a revisit. Reports.

[3] Wang M., Liao Z. (2020). SARS-CoV-2 and COVID-19: how much do we know?. Acta Virol.

[4] Fu Y., Wang M., Zhao B. (2021). Psychological impact of COVID-19 cases on medical staff of Beijing Xiaotangshan Hospital. Psychol Res Behav Manag.

[5] Nardo A.D., Schneeweiss-Gleixner M., Bakail M., Dixon E.D., Lax S.F., Trauner M. (2021). Pathophysiological mechanisms of liver injury in COVID-19. Liver Int.

[6] Zhao X., Lei Z., Gao F., Xie Q., Jang K., Gong J. (2021). The impact of coronavirus disease 2019 (COVID-19) on liver injury in China: a systematic review and meta-analysis. Medicine (Baltimore).

[7] Zhang C., Shi L., Wang F.S. (2020). Liver injury in COVID-19: management and challenges. Lancet Gastroenterol Hepatol.

[8] Tian D., Ye Q. (2020). Hepatic complications of COVID-19 and its treatment. J Med Virol.

[9] Dawood D.R.M., Salum G.M., El-Meguid M.A. (2022). The impact of COVID-19 on liver injury. Am J Med Sci.

[10] Guan W.J., Ni Z.Y., Hu Y. (2020). Clinical characteristics of coronavirus disease 2019 in China. N Engl J Med.

[11] Mao R., Qiu Y., He J.S. (2020). Manifestations and prognosis of gastrointestinal and liver involvement in patients with COVID-19: a systematic review and meta-analysis. Lancet Gastroenterol Hepatol.

[12] Henry B.M., de Oliveira M.H.S., Benoit S., Plebani M., Lippi G. (2020). Hematologic, biochemical and immune biomarker abnormalities associated with severe illness and mortality in coronavirus disease 2019 (COVID-19): a meta-analysis. Clin Chem Lab Med.

[13] Alqahtani S.A., Schattenberg J.M. (2020). Liver injury in COVID-19: the current evidence. United European Gastroenterol J.

[14] Dong M., Zhang J., Ma X. (2020). ACE2, TMPRSS2 distribution and extrapulmonary organ injury in patients with COVID-19. Biomed Pharmacother.

[15] Schattenberg J.M., Labenz C., Wörns M.A. (2020). Patterns of liver injury in COVID-19 - a German case series. United European Gastroenterol J.

[16] Lucas S. (2022). Where does SARS-CoV-2 go to in man?^†^. J Pathol.

[17] Yele V., Sanapalli B.K.R., Mohammed A.A. (2022). Imidazoles and benzimidazoles as putative inhibitors of SARS-CoV-2 B.1.1.7 (Alpha) and P.1 (Gamma) variant spike glycoproteins: a computational approach. Chem Zvesti.

[18] Liu C., Zhou D., Nutalai R. (2022). The antibody response to SARS-CoV-2 Beta underscores the antigenic distance to other variants. Cell Host Microbe.

[19] Moss D.L., Rappaport J. (2021). SARS-CoV-2 beta variant substitutions alter spike glycoprotein receptor binding domain structure and stability. J Biol Chem.

[20] Sanches P.R.S., Charlie-Silva I., Braz H.L.B. (2021). Recent advances in SARS-CoV-2 Spike protein and RBD mutations comparison between new variants Alpha (B.1.1.7, United Kingdom), Beta (B.1.351, South Africa), Gamma (P.1, Brazil) and Delta (B.1.617.2, India). J Virus Erad.

[21] Grishin A.M., Dolgova N.V., Landreth S. (2022). Disulfide bonds play a critical role in the structure and function of the receptor-binding domain of the SARS-CoV-2 spike antigen. J Mol Biol.

[22] Chen Y., Guo Y., Pan Y., Zhao Z.J. (2020). Structure analysis of the receptor binding of 2019-nCoV. Biochem Biophys Res Commun.

[23] Qi F., Qian S., Zhang S., Zhang Z. (2020). Single cell RNA sequencing of 13 human tissues identify cell types and receptors of human coronaviruses. Biochem Biophys Res Commun.

[24] Peacock T.P., Goldhill D.H., Zhou J. (2021). The furin cleavage site in the SARS-CoV-2 spike protein is required for transmission in ferrets. Nat Microbiol.

[25] Rando H.M., MacLean A.L., Lee A.J. (2021). Pathogenesis, symptomatology, and transmission of SARS-CoV-2 through analysis of viral genomics and structure. mSystems.

[26] Tai L., Zhu G., Yang M. (2021). Nanometer-resolution in situ structure of the SARS-CoV-2 postfusion spike protein. Proc Natl Acad Sci U S A.

[27] Bharadwaj S., Singh M., Kirtipal N., Kang S.G. (2021). SARS-CoV-2 and glutamine: SARS-CoV-2 triggered pathogenesis via metabolic reprograming of glutamine in host cells. Front Mol Biosci.

[28] Desquilles L., Cano L., Ghukasyan G. (2022). Well-differentiated liver cancers reveal the potential link between ACE2 dysfunction and metabolic breakdown. Sci Rep.

[29] Song L.N., Liu J.Y., Shi T.T. (2020). Angiotensin-(1-7), the product of ACE2 ameliorates NAFLD by acting through its receptor Mas to regulate hepatic mitochondrial function and glycolipid metabolism. FASEB J.

[30] Galaris D., Barbouti A., Pantopoulos K. (2019). Iron homeostasis and oxidative stress: an intimate relationship. Biochim Biophys Acta Mol Cell Res.

[31] Mercado-Gómez M., Prieto-Fernández E., Goikoetxea-Usandizaga N. (2022). The spike of SARS-CoV-2 promotes metabolic rewiring in hepatocytes. Commun Biol.

[32] Auriti C., De Rose D.U., Santisi A. (2021). Pregnancy and viral infections: mechanisms of fetal damage, diagnosis and prevention of neonatal adverse outcomes from cytomegalovirus to SARS-CoV-2 and Zika virus. Biochim Biophys Acta Mol Basis Dis.

[33] Chen N., Zhou M., Dong X. (2020). Epidemiological and clinical characteristics of 99 cases of 2019 novel coronavirus pneumonia in Wuhan, China: a descriptive study. Lancet.

[34] Hojyo S., Uchida M., Tanaka K. (2020). How COVID-19 induces cytokine storm with high mortality. Inflamm Regen.

[35] Yu Y., Liu Y., An W., Song J., Zhang Y., Zhao X. (2019). STING-mediated inflammation in Kupffer cells contributes to progression of nonalcoholic steatohepatitis. J Clin Invest.

[36] Idalsoaga F., Ayares G., Arab J.P., Díaz L.A. (2021). COVID-19 and indirect liver injury: a narrative synthesis of the evidence. J Clin Transl Hepatol.

[37] Napodano C., Pocino K., Stefanile A. (2021). COVID-19 and hepatic involvement: the liver as a main actor of the pandemic novel. Scand J Immunol.

[38] Adams D.H., Hubscher S.G. (2006). Systemic viral infections and collateral damage in the liver. Am J Pathol.

[39] Meskar A., Plee-Gautier E., Amet Y., Berthou F., Lucas D. (2001). Alcohol-xenobiotic interactions. Role of cytochrome P450 2E1. Pathol Biol (Paris).

[40] Wang M., Liu L., Zhang C.S. (2020). Mechanism of traditional Chinese medicine in treating knee osteoarthritis. J Pain Res.

[41] Zha B.S., Wan X., Zhang X. (2013). HIV protease inhibitors disrupt lipid metabolism by activating endoplasmic reticulum stress and inhibiting autophagy activity in adipocytes. PLoS One.

[42] Cao R., Hu Y., Wang Y. (2010). Prevention of HIV protease inhibitor-induced dysregulation of hepatic lipid metabolism by raltegravir via endoplasmic reticulum stress signaling pathways. J Pharmacol Exp Ther.

[43] Ferrara F., Granata G., Pelliccia C., La Porta R., Vitiello A. (2020). The added value of pirfenidone to fight inflammation and fibrotic state induced by SARS-CoV-2: anti-inflammatory and anti-fibrotic therapy could solve the lung complications of the infection?. Eur J Clin Pharmacol.

[44] Jaeschke H. (2015). Acetaminophen: dose-dependent drug hepatotoxicity and acute liver failure in patients. Dig Dis.

[45] Saheb Sharif-Askari N., Saheb Sharif-Askari F., Mdkhana B. (2020). Effect of common medications on the expression of SARS-CoV-2 entry receptors in liver tissue. Arch Toxicol.

[46] Jin C., Lin L., Han N. (2021). Risk of gestational diabetes mellitus in relation to plasma concentrations of fatty acid-binding protein 4: a nested case-control study in China. J Diabetes Res.

[47] Kumar-M P., Mishra S., Jha D.K. (2020). Coronavirus disease (COVID-19) and the liver: a comprehensive systematic review and meta-analysis. Hepatol Int.

[48] Marjot T., Webb G.J., Barritt A.S. 4th (2021). COVID-19 and liver disease: mechanistic and clinical perspectives. Nat Rev Gastroenterol Hepatol.

[49] Lei P., Zhang L., Han P. (2020). Liver injury in patients with COVID-19: clinical profiles, CT findings, the correlation of the severity with liver injury. Hepatol Int.

[50] Imoto K., Kohjima M., Hioki T. (2019). Clinical features of liver injury induced by immune checkpoint inhibitors in Japanese patients. Can J Gastroenterol Hepatol.

[51] Di Sessa A., Lanzaro F., Zarrilli S. (2021). COVID-19 and pediatric fatty liver disease: is there interplay?. World J Gastroenterol.

[52] Marjot T., Moon A.M., Cook J.A. (2021). Outcomes following SARS-CoV-2 infection in patients with chronic liver disease: an international registry study. J Hepatol.

[53] Lebossé F., Gudd C., Tunc E. (2019). CD8^+^T cells from patients with cirrhosis display a phenotype that may contribute to cirrhosis-associated immune dysfunction. EBioMedicine.

[54] Arvaniti V., D’Amico G., Fede G. (2010). Infections in patients with cirrhosis increase mortality four-fold and should be used in determining prognosis. Gastroenterology.

[55] Marjot T., Webb G.J., Barritt A.S. (2021). SARS-CoV-2 vaccination in patients with liver disease: responding to the next big question. Lancet Gastroenterol Hepatol.

[56] Boeckmans J., Rodrigues R.M., Demuyser T., Piérard D., Vanhaecke T., Rogiers V. (2020). COVID-19 and drug-induced liver injury: a problem of plenty or a petty point?. Arch Toxicol.

[57] Yang R.X., Zheng R.D., Fan J.G. (2020). Etiology and management of liver injury in patients with COVID-19. World J Gastroenterol.

[58] Meng M., Chu Y., Zhang S. (2022). Corticosteroid treatment in severe patients with SARS-CoV-2 and chronic HBV co-infection: a retrospective multicenter study. BMC Infect Dis.

[59] Amaddeo G., Brustia R., Allaire M. (2021). Impact of COVID-19 on the management of hepatocellular carcinoma in a high-prevalence area. JHEP Rep.

[60] Ritschl P.V., Nevermann N., Wiering L. (2020). Solid organ transplantation programs facing lack of empiric evidence in the COVID-19 pandemic: a By-proxy Society Recommendation Consensus approach. Am J Transplant.

[61] Phipps M.M., Barraza L.H., LaSota E.D. (2020). Acute liver injury in COVID-19: prevalence and association with clinical outcomes in a large U.S. cohort. Hepatology.

[62] Del Valle D.M., Kim-Schulze S., Huang H.H. (2020). An inflammatory cytokine signature predicts COVID-19 severity and survival. Nat Med.

[63] Colmenero J., Rodríguez-Perálvarez M., Salcedo M. (2021). Epidemiological pattern, incidence, and outcomes of COVID-19 in liver transplant patients. J Hepatol.

[64] Webb G.J., Marjot T., Cook J.A. (2020). Outcomes following SARS-CoV-2 infection in liver transplant recipients: an international registry study. Lancet Gastroenterol Hepatol.

[65] Sahin T.T., Akbulut S., Yilmaz S. (2020). COVID-19 pandemic: its impact on liver disease and liver transplantation. World J Gastroenterol.

[66] Ng A. (2021). Liver injury in liver transplant patients with COVID-19: a histopathologic analysis. Clin Gastroenterol Hepatol.

[67] McCashland T.M., Preheim L.C., Gentry M.J. (2000). Pneumococcal vaccine response in cirrhosis and liver transplantation. J Infect Dis.

[68] Saklayen M.G. (2018). The global epidemic of the metabolic syndrome. Curr Hypertens Rep.

[69] Khan R.M.M., Chua Z.J.Y., Tan J.C., Yang Y., Liao Z., Zhao Y. (2019). From pre-diabetes to diabetes: diagnosis, treatments and translational research. Medicina (Kaunas).

[70] Phua W.W.T., Wong M.X.Y., Liao Z., Tan N.S. (2018). An aPPARent functional consequence in skeletal muscle physiology via peroxisome proliferator-activated receptors. Int J Mol Sci.

[71] Wang M., Yang Y., Liao Z. (2020). Diabetes and cancer: epidemiological and biological links. World J Diabetes.

[72] Wang M., Tan Y., Shi Y., Wang X., Liao Z., Wei P. (2020). Diabetes and sarcopenic obesity: pathogenesis, diagnosis, and treatments. Front Endocrinol (Lausanne).

[73] Chi T., Lin J., Wang M., Zhao Y., Liao Z., Wei P. (2021). Non-coding RNA as biomarkers for type 2 diabetes development and clinical management. Front Endocrinol (Lausanne).

[74] Lin J.R., Wang Z.T., Sun J.J. (2022). Gut microbiota and diabetic kidney diseases: pathogenesis and therapeutic perspectives. World J Diabetes.

[75] Li R., Tang Y., Liang M., Ding J. (2021). Liver injury in COVID-19 patients with metabolic syndrome-a narrative review. Ann Palliat Med.

[76] Shi Y., Wu L.Q., Wei P., Liao Z.H. (2022). Children with type 1 diabetes in COVID-19 pandemic: difficulties and solutions. World J Clin Pediatr.

[77] Pellegrini M., Ponzo V., Rosato R. (2020). Changes in weight and nutritional habits in adults with obesity during the “lockdown” period caused by the COVID-19 virus emergency. Nutrients.

[78] Yang G., Tan Z., Zhou L. (2020). Effects of angiotensin II receptor blockers and ACE (angiotensin-converting enzyme) inhibitors on virus infection, inflammatory status, and clinical outcomes in patients with COVID-19 and hypertension: a single-center retrospective study. Hypertension.

[79] Lu Y., Liu D.X., Tam J.P. (2008). Lipid rafts are involved in SARS-CoV entry into Vero E6 cells. Biochem Biophys Res Commun.

[80] Guzmán-Flores J.M., López-Briones S. (2012). Cells of innate and adaptive immunity in type 2 diabetes and obesity. Gac Med Mex.

[81] Portincasa P., Krawczyk M., Smyk W., Lammert F., Di Ciaula A. (2020). COVID-19 and non-alcoholic fatty liver disease: two intersecting pandemics. Eur J Clin Invest.

[82] Nave H., Beutel G., Kielstein J.T. (2011). Obesity-related immunodeficiency in patients with pandemic influenza H1N1. Lancet Infect Dis.

[83] Omarjee L., Janin A., Perrot F., Laviolle B., Meilhac O., Mahe G. (2020). Targeting T-cell senescence and cytokine storm with rapamycin to prevent severe progression in COVID-19. Clin Immunol.

[84] Mishra K., Naffouj S., Gorgis S. (2020). Liver injury as a surrogate for inflammation and predictor of outcomes in COVID-19. Hepatol Commun.

[85] Jiang B., Wei H. (2020). Oxygen therapy strategies and techniques to treat hypoxia in COVID-19 patients. Eur Rev Med Pharmacol Sci.

[86] Cichoz-Lach H., Michalak A. (2021). Liver injury in the era of COVID-19. World J Gastroenterol.

[87] Pereira M.R., Mohan S., Cohen D.J. (2020). COVID-19 in solid organ transplant recipients: initial report from the US epicenter. Am J Transplant.

[88] Li S., Li J., Zhang Z. (2020). COVID-19 induced liver function abnormality associates with age. Aging (Albany NY).

[89] Fan Z., Chen L., Li J. (2020). Clinical features of COVID-19-related liver functional abnormality. Clin Gastroenterol Hepatol.

[90] Chen L., Li Q., Zheng D. (2020). Clinical characteristics of pregnant women with Covid-19 in Wuhan, China. N Engl J Med.

[91] Deng G., Zeng F., Zhang L., Chen H., Chen X., Yin M. (2020). Characteristics of pregnant patients with COVID-19 and liver injury. J Hepatol.

[92] Can E., Oğlak S.C., Ölmez F. (2022). Abnormal liver function tests in pregnant patients with COVID-19 - a retrospective cohort study in a tertiary center. Ginekol Pol.

[93] Yadav D.K., Singh A., Zhang Q. (2021). Involvement of liver in COVID-19: systematic review and meta-analysis. Gut.

[94] Ali N., Hossain K. (2020). Liver injury in severe COVID-19 infection: current insights and challenges. Expert Rev Gastroenterol Hepatol.

